# Bridges or Barriers? Conceptualization of the Role of Multiple Identity Gateway Groups in Intergroup Relations

**DOI:** 10.3389/fpsyg.2017.01097

**Published:** 2017-06-29

**Authors:** Aharon Levy, Tamar Saguy, Eran Halperin, Martijn van Zomeren

**Affiliations:** ^1^The School of Psychology, Interdisciplinary Center HerzliyaHerzliya, Israel; ^2^Heymans Institute for Psychological Research, University of GroningenGroningen, Netherlands

**Keywords:** multiple identity, gateway groups, intergroup conflict, conflict resolution, dual identity, cross categorization, biracial, social identity complexity

## Abstract

The modern era of globalization has been accompanied by a massive growth in interconnections between groups, and has led to the sharing of multiple identities by individuals and groups. Following these developments, research has focused on the issue of multiple identities, and has shed important light on how *individuals* who hold these complex forms of identity feel and behave, and on the reactions they elicit from members of other groups. However, the potential of *groups* with such multiple identities (e.g., biracials, immigrants, etc.) to affect the intergroup relations between the groups that represent the respective sources of the different identities (e.g., Blacks and Whites, country of origin and country of residence, etc.) has not been examined to date. Accordingly, in this paper, we first systematically explore the potential of groups in which people identify with multiple social categories, or groups that are perceived as such by others, to play a role in intergroup dynamics. Next, we offer a theoretical framework outlining what functions groups of people with shared multiple identities may serve (as *bridges* or *barriers*) by proposing how their presence may facilitate or deteriorate intergroup relations. Finally, we present recent empirical research examining how groups of people with shared multiple identities can act as *gateways* and bridge the cleft between two separate groups that represent the respective sources of their different identities, and discuss the theoretical and practical implications for the field of intergroup relations.

## Introduction

Race, at least in the United States, has typically been treated as a dichotomy (e.g., Black or White), with individuals challenging this racial dichotomy being likely to become socially excluded or even penalized (Hickman, 1997; Davis, 2010; Khanna, 2010; Wagner et al., 2010). However, in recent years a clear shift seems to be taking place toward an increase in both the presence and influence of biracial identity. Indeed, over the past 15 years, the Black and White biracial population in the United States has tripled in size numbering over 2.5 million (U.S. Bureau of the Census, 2015, based on self-report), and the current estimate is that by 2050 one out of five Americans will be of mixed-race (Lee and Bean, 2004). Indeed, recent research even suggests the rise of a new multi-racial identity that is replacing the monolithic race identities in the United States census (Roth, 2005; Davenport, 2016). The million-dollar question is how these identity developments will affect the existing intergroup relations between Blacks and Whites in the United States, and how biracials will be perceived by others, and perceive themselves, now that they are becoming more and more a prevalent in social reality.

This million-dollar question, however, is not limited solely to the realm of race. Just as biracials are strategically situated between Blacks and Whites on a structural level of analysis, so are, for example, the Bosnian citizens of Serbia situated in the same kind of social overlap between Serbia and Bosnia. Thus, this group which has a mixture of Bosnian and Serbian national identities, may have similar influence on the international social dynamics between the two nations in conflict. Moreover, multiple identities exist not only in the same dimension (biracial, dual nationality), but also as a result of cross cutting identities such as nationality and religion. For example, the Muslim community in the Kashmir region of India share their national identity with India while at the same time share their religious identity with Pakistan, and as such may be able to affect the relations between these two conflicting countries as well. In fact, similar social structures can be found in many other contexts and levels of analysis as well (which will be elaborated on below), raising the same question of how the emergence of such a group with a mixed identity will influence intergroup relations, and what this implies for members of groups with such multiple identities.

This question of how multiple social identities affect intergroup relations remains largely unanswered by contemporary social psychology, mainly because research seems to have focused on how individuals who hold these complex forms of categorization feel and behave (Baysu et al., 2011; Gocłowska and Crisp, 2014), and on the reactions they elicit from members of the dominant group (González and Brown, 2006; Bodenhausen, 2010; Rodeheffer et al., 2012; Scheepers et al., 2014; Urbiola et al., 2017). This research has found for example, that multiple identification among minority groups (e.g., both German *and* Turkish) typically relates to better well-being (Sam and Berry, 2010; Nguyen and Benet-Martínez, 2013), and that for majority group members, endorsing the existence of multiple identities (as reflected in the notion of multiculturalism) promotes more positive outgroup attitudes (Wolsko et al., 2000; Scheepers et al., 2014).

However, despite the importance of these findings, they leave several key questions unanswered. More specifically, we seem to know relatively little about the potential of groups equipped with multiple identities to affect the intergroup relations between the groups that represent the respective sources of their multiple identities. Given their shared identity with both groups, groups of people with shared multiple identities can potentially *bridge* the cleft between two (or more) otherwise separate groups (e.g., if someone is your sister’s sister than she is probably your sister as well). At the same time, under certain conditions these groups of people with shared multiple identities can also pose *barriers* to conflict resolution by raising issues of trust and betrayal. The main goals of this paper are therefore to outline the notion of such *gateway groups* (GGs) amidst intergroup conflict, and to situate it in a novel theoretical framework. This framework will outline what functions GGs may serve (as *bridges* or *barriers*), and how these functions may improve or deteriorate intergroup conflict both among those who perceive these groups from the outside, as well as among the members of the GGs themselves. Where relevant, we will further review data supporting elements of the theoretical framework proposed.

Of course, this is not just important theoretically, but also for practical reasons. When considering the potential positive effect of groups of people with shared multiple identities, Turkish immigrants in Germany for example, may be able to impact the relations between Turks and Germans in general by virtue of being perceived as identifying with both these entities. Similarly, biracials in the United States might have the ability to bridge relations between Blacks and Whites, and Arab citizens of Israel might likewise be able to influence the relations between Israel and Palestine. Thus, our approach focuses on the psychological perceptions, experience, and functions of GGs, both from the perspective of the groups in conflict and of the GG members themselves.

Despite the potential of GGs for improving intergroup relations, GGs might also serve as *barriers* as much as *bridges*, for instance when GG members feel their multiple identities to be in conflict with each other, or when groups in conflict do not trust GG members because they fear mixed and even shifting loyalties. This possible complexity may lead to a decrease in GG members’ dual identification. At worst, the GG might even be perceived as a “fifth column” that undermines the position of either group in the broader conflict. This may have negative consequences for intergroup conflict in general, and presumably also for GG members’ well-being.

Therefore, in this paper we will map the existing findings regarding cases in which the GGs serve their *bridging* function (which improves intergroup relations and has positive psychological consequences for all involved), and cases in which they serve their *barrier* function (which deteriorates intergroup relations and has negative psychological consequences for all involved). Below, we first define and conceptualize GGs, and review recent evidence regarding the positive and negative potential of such groups when their members *perceive themselves* as having multiple identities. Then, we present recent data for the positive potential of such groups *being perceived* as having multiple identities and consider possible setbacks that this may have. Finally, we discuss functions of GGs that have not been examined and develop relevant hypotheses.

## Definition and Conceptualization

We define GGs as groups characterized by unique social categorizations that enables them to be categorized as and identified with more than one group within the context of intergroup relations. Importantly, the categorization of a group as a GG can stem from the way others *perceive* this group, or by how the group members *experience* their own group, or both. Due to the fractal nature of social categorization that can be analyzed on several different levels of analysis, there can be many different types of GGs. As mentioned above, GGs can exist on a national level (e.g., Israeli Arabs) and on a racial level (e.g., biracials). Additionally, GGs can be found on a larger global cultural level, countries such as Turkey or Albania can mediate between the western world, and the Muslim world which they are both identified with (Keyman, 2007); and on a smaller scale situated in the midst of a specific ethnic group (e.g., between Ashkenazi and Sephardic Jews; see Cohen et al., 2004).

## GG and Existing Multiple Identity Literature

The GG concept fits smoothly within the existing literature on multiple identities, although a significant distinction that can be made here is that of *perspective*. While existing frameworks are mainly rooted in the perspective of the dual or multiple identifier (i.e., focusing on how individuals cope with multiple identities), the GG concept can also refer to the perspective of social groups that view the GG from the outside. For instance, if the biracial community is perceived by the White community and the Black community as biracial, then the biracial GG may have an impact on this intergroup relation even if the biracial individuals do not necessarily subjectively identify with both groups simultaneously (and vice versa). This conceptualization enables us to study both perspectives systematically. Thus, we introduce a clear distinction between the type of perspective on the groups of people with shared multiple identities (i.e., does existing research deal with how these groups are perceived by others, or does it deal with how the members of these groups perceive themselves).

Furthermore, multiple identities can have different functions in the contexts of intergroup relations (i.e., as *bridges* or *barriers*) that have either positive or negative effects on the relations between the groups that represent the sources of those identities. The suggested framework is a 2 × 2 matrix based on the perspective on the groups of people with shared multiple identities (from within or without), and on the function of the groups of people with shared multiple identities (bridge or barrier)^1^.

## Positive Outcome of Group Members *Perceiving Themselves* as Having Multiple Identities

Several studies have examined the positive outcomes that stem from individuals’ identification with multiple social groups. Theory and research on the so-called *social cure* contends that identifying with multiple social groups is directly linked to improved health and well-being (Jetten et al., 2012, 2014; Steffens et al., 2016a,b). However, since our focus in this paper is on the function of multiple identities in the context of intergroup relations, we will focus specifically on the possible outcomes that stem from multiple identities and affect intergroup relations. In this section, we will briefly review relevant work done on dual identity, common ingroup identity, cross-categorization, and social identity complexity, and describe how these lines of thought explain the positive outcome of multiple identities in intergroup relations.

### Dual Identity

Arguably the most relevant line of work for the present purposes is theory and research on *dual identity* (Brown and Hewstone, 2005; Dovidio et al., 2009). Dual identity is a simultaneous identification with a distinct subgroup and a common superordinate group (e.g., the Latino minority in the United States that identifies simultaneously as Latino and as American; Dovidio et al., 2009). The central benefit of dual identification lies in allowing minority group members to feel connected to the dominant majority group, while maintaining their distinctiveness as a separate group simultaneously. In several empirical studies, this dual identification was found to be associated with numerous constructive outcomes including the well-being of the dual identifiers (Sam and Berry, 2010; Nguyen and Benet-Martínez, 2013), and inhibition of extremism (Simon and Ruhs, 2008). Such outcomes are typically explained by the notion that they fulfill individuals’ need to identify with their original subgroup while still feeling connected to a larger whole (Brewer, 1991).

Additionally, following the logic of the *common ingroup identity model* (Gaertner et al., 1993), dual identities might signal to the respective groups that a superordinate identity, incorporating both groups, is possible. Such a common identity has been found as a useful tool for the reduction of intergroup prejudice. While a common ingroup identity might also have negative effects on disadvantaged groups, and seems very hard to maintain in the context of intergroup conflict (Saguy et al., 2009), the fleshing out of dual identity aspects can help maintain such a superordinate identity while counteracting the possible negative side effects (Dovidio et al., 2009). Thus, dual identity can induce the positive impact of a common group identity despite the described difficulties. For example, in a study by Hornsey and Hogg (2000), when university students were primed with both their superordinate identity (university students), and their subordinate identity (humanities or math-science students) simultaneously, they displayed lower levels of bias toward their outgroup compared to when they were primed only with their superordinate or their subordinate identities separately. As we will later elaborate, the notion of GGs resonates with this dual identity construct in that the presence of a multiple identity GG can be both a reminder for its counterparts of the similarity between the two separate groups, while at the same time help maintain each group’s distinction.

An interesting example of the potential of a dual identity when paired with a common ingroup identity can be found in a few recent studies examining collective action tendencies among disadvantaged-group members in the United States (Blacks and Latinos, see for example Ufkes et al., 2016). These studies have found that increasing only the salience of a common United States identity among Blacks and Latinos reduced intergroup bias but also resulted in lower collective action intentions, and thus led to the maintenance of existing social inequality. Increasing salience of dual identity, however, did not decrease collective action intentions, and was found to both reduce intergroup bias as well as challenge existing social inequality.

### Social Categorization Overlap

Another very rich source of information on the positive implications of individuals and groups perceiving themselves as having multiple identities is in the field of social category overlap. The work on *cross-categorization*, for example, addresses the multiple identities an individual holds, and their potential overlap. According to research on cross-categorization, given that individuals are members of several groups simultaneously (e.g., both Black and female), members of an outgroup on one dimension may be evaluated more positively if they are also ingroup members on another dimension (e.g., a Black women evaluating a White woman). This crossing of categories was found to reduce intergroup prejudice and discrimination among those holding the multiple identities (Brewer and Campbell, 1976; Deschamps and Doise, 1978; Migdal et al., 1998; Crisp and Hewstone, 1999; Hutter and Crisp, 2005).

Similarly, *social identity complexity* also deals with the overlap between different social identities (specifically the extent of such overlap) and posits that raising awareness to the partiality of overlap between social identities decreases the salience of social categories, and in turn raises tolerance for outgroups in general (Roccas and Brewer, 2002; Brewer and Pierce, 2005; Brewer, 2010; Branković, 2016; Sønderlund et al., 2017). For example, in a study by Vasiljevic and Crisp (2013), participants who were primed with a multiple social categorization mindset, increased cognitive flexibility, displayed lowered prejudice toward a multitude of outgroups, fostered egalitarian values, and enhanced their trust toward outgroups. Here too, the GG notion can be seen as building on this existing construct, as the GGs are inherently characterized by identity overlap as we will describe below.

In sum, the literature described above, which is a culmination of decades of studying multiple identities in intergroup contexts, provides clear indication that when it comes to intergroup relations there are several positive functions multiple identification may have on those who hold it. Whether in facilitating a common superordinate identity, increasing cognitive flexibility, or reducing intergroup prejudice, both the multiple identifiers and their counterparts stand to gain from these groups identifying themselves with more than one social category. Having said that, we also believe that it is important to consider the potentially *negative* side of GGs’ multiple identification, and asses the possible backlash of groups perceiving themselves as having multiple identities in the context of intergroup relations.

## Negative Outcome of Group Members *Perceiving Themselves* as Having Multiple Identities

Despite the promising potential of GGs in terms of group members perceiving themselves as having multiple identities (as described above), there is an important reservation to make in this regard, and that is the possible backlash the fleshing out of such multiple identities may have in the context of intergroup relations. In terms of individuals perceiving themselves as having multiple identities, a negative side effect that can possibly result from stressing the multiple identification of a group, can be the decreased well-being amongst group members themselves (Gaither et al., 2013). Some of the research in the field of dual and multiple identities have found that there can be a downside to holding multiple identities, especially in the context of intergroup conflict.

For example, in cases where intergroup relations are tense, multiple identifiers are sometimes forced to distance themselves from one of their ingroups, and even partake in ingroup derogation in the attempt to overcome disadvantage in cases of social inequality (Verkuyten and Reijerse, 2008; Derks et al., 2015, 2016; Kulich et al., 2015). Moreover, under some conditions, biracial individuals may feel anxiety that stems from interracial encounters while encountering both Blacks and Whites (Gaither et al., 2013).

Furthermore, when the identities multiple identifiers hold are perceived as incompatible, it may foster controversial or even destructive forms of political radicalism (Simon et al., 2013). Finally, research on dual identifiers in the context of intergroup conflict has shown, that the tension between the conflicting sources of the dual identity causes the dual identifiers to be marginalized by both counterparts. This double marginality may lead to the general exclusion of these groups of people with shared multiple identities, weaken their collective infrastructure, and at times even bring about collective identity crisis (Al-Haj, 2000; Berdahl and Moore, 2006; Lowrance, 2006; Pinson, 2008). Another relevant notion in this regard is that of *intersectionality*, which claims that individuals’ identification with more than one discriminated group in an intergroup context may lead to marginalization of wrongdoings toward such a group, even compared to other discriminated groups that do not have multiple identities (Crenshaw, 1991). Although this is not a direct negative outcome of holding multiple identities, it only applies to those who hold more than one discriminated social identity, and thus also corresponds with the framework we are suggesting.

In sum, despite the positive potential of GGs holding multiple identities in the context of intergroup relations, this element also has the potential of putting such GGs between the proverbial identity rock and hard place. This suggests that perceiving oneself as having multiple identities might not always be so beneficial, and this may also be the case when being perceived as such by external groups.

## Positive Outcome of Groups *Being Perceived* as Having Multiple Identities

As important as it is to discover the outcomes of individuals’ identification with multiple groups in the context of intergroup relations, it is equally important to understand the potential of the presence of GGs to affect the intergroup relations between the groups that represent the respective sources of their multiple identities. Even though, to our knowledge, no prior work has taken the approach we propose here with respect to the potential role of GGs in intergroup conflicts, the work described above on social categorization processes lays the groundwork and intrinsically corresponds with the GG notion we will describe below.

For instance, the presence of a multiple identity GG can be both a reminder for its counterparts of the similarity between the two separate groups, and a signifier of the group’s distinction (Saguy et al., 2009). As such, multiple identity GGs can be utilized in order to highlight dual identities and foster common ingroup identities among those who perceive the GG as holding multiple identities. Additionally, since GGs can be seen as social groups in which the ingroup identity overlaps with the outgroup identity, the research on cross-categorization is highly relevant as well. The effects of intergroup prejudice reduction that cross categorization was found to have among group members that perceive their own identities as crisscrossing, may also take place in scenarios involving others that perceive the GGs as holding cross-cutting identities. Given that little is known about such scenarios, we present our line of thought below, and provide some first evidence of the positive potential that the presence of GGs can have in intergroup conflict.

### Extending the Existing Research

Besides the direct links between the GG notion and existing literature described above, in which this notion can be seen as an integral part of existing work, the GG notion can theoretically broaden the existing scope of the multiple identity literature as well. First, while dual identity has primarily been linked with *hierarchically nested* identities in the form of a superordinate (typically majority) and the subgroup identity (typically minority group. e.g., Turkish immigrants in Germany that are nested in the superordinate German identity while maintaining a separate Turkish identity), GGs also incorporates situations in which identities are not nested. For example, the biracial community in the United States. Does not necessarily have a clear hierarchically nested structure between its White and Black identity (i.e., neither racial identity encompasses the other). Thus, all dual identifiers can be seen as members of a GGs but not all GGs can be explained with the notion of dual identity.

Second, dual identification scenarios usually include three different social agents: two distinct social groups, and the dual-identity group. However, to our knowledge the existing literature only address two of these social agents: one of the two distinct groups, and the dual identifiers. For example, in the case of the Mexican minority in the United States. Most of the literature addresses either the minority itself, or the White Americans and their interaction with the Mexican minority, but the Mexicans in Mexico are not addressed. The broader notion of a GG enables the incorporation of several different relevant groups for a more inclusive, realistic, and complex understanding of intergroup dynamics. For example, due to the fact that the GG viewpoint accounts for all three parties in this scenario, it would enable the explication of phenomena such as United States presidential candidates courting Mexican officials during a United States presidential campaign, as well as a more accurate modeling of the intergroup dynamic between Mexicans and Americans inside and outside of the United States.

**FIGURE 1:**
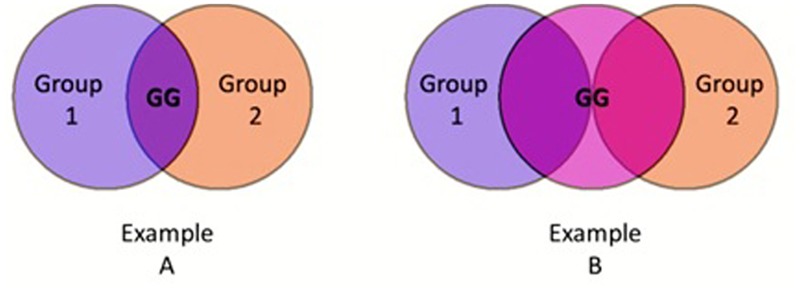
Two possible GG scenario depictions. In example A there is a perceived overlap between the two social categories and the GG consists of the group members situated in that overlap. On the other hand, in example B, the perception is not of a social category overlap between the two distinct groups, but of an overlap that both groups have with a shared GG.

Finally, whereas cross categorization deals with meshing identities from different dimensions (e.g., race and sex), GGs create an overlap between identities from a single dimension (e.g., the overlap of two different racial groups). Thus, the GG fills an important gap not fully covered by cross categorization, of the identity overlap between identities from the same dimension such as national identities overlapping in immigrant communities, or racial identities overlapping among biracials. Moreover, cross categorization usually requires a positive overlap between two identities in order to take effect (see example A in **Figure 1**). However, from an external perspective the existence of a GG might suffice in order to achieve the positive effects of cross categorization in scenarios not deemed eligible in the past, by creating an identity overlap outside of the conflicting parties and inside the GG itself (see example B in **Figure 1**). Take for instance, the intergroup relations between religious people and gay people. These two communities conceptually do not overlap, and therefore are not a natural candidate for cross-categorization. Nonetheless, the existence of a religious and gay community GG might be able to symbolize the necessary overlap needed to induce the positive cross categorization effect for both of these respective groups.

Taken together, the existing research on multiple identification can be used to predict that the presence of a GG may lead to positive outgroup orientations also among external groups that perceive it as such. Moreover, this may also enable the broadening of the dual identity prospect as described above, and extend the explanatory scope of the multiple identities literature as well. Based on this assumption we designed several studies in order to examine the potential of multiple identity GGs to improve intergroup relations between the external groups representing the respective sources of the multiple GG identities. Thus, we studied how others perceive those in a multiple identity GG, and how that perception influences intergroup relations (Levy et al., 2017, under review).

### Perceived GG Empirical Studies Overview

In one recent paper (Levy et al., 2017), we first conducted a pilot study designed to simply compare the perceptions and attitudes toward an outgroup to the perceptions and attitudes toward a GG. Studies have found that under certain conditions biracials maybe simply perceived as Black, i.e., distinct outgroup, and not in the complex manner we have described (Peery and Bodenhausen, 2008; Rodeheffer et al., 2012). Accordingly, this study was meant to examine the premise that GGs with multiple identities are indeed perceived differently and more positively (or less negatively) than the distinct outgroup. The study was conducted in the Israeli-Palestinian conflict context among Israeli participants (ingroup), with the Palestinians as the outgroup, and the Arab citizens of Israel as the GG. This is a good example of a multiple identity GG because Arab citizens of Israel are affiliated with both the Israeli Jewish group with whom they share their citizenship as well as with the Palestinian group residing in the Westbank and Gaza, with whom they share their national identity, and thus can serve as a GG. As expected, the GG was evaluated more positively than the outgroup in almost every single indicator including: group stereotypes, perceived similarity with the ingroup, contact motivation with group members, support for aggression against the group, and feelings of anger and hate toward the group. These findings confirmed our assumption regarding the positive potential of GGs for improving intergroup relations. However, these findings may be restricted to this particular context, and needed replication (to safeguard external validity) and corroboration in terms of internal validity (through an experimental approach).

Therefore, the next study in this line of research was a correlational study meant to examine the correlation between the perception of the GG as holding multiple identities, and positive attitudes between the groups reflected in the GG. Based on the GG hypothesis, the positive attitudes between the groups that made up the multiple identities should be higher when they indeed viewed the intermediate GG as having multiple identities. To facilitate external validity, the correlational study was run in two separate contexts: The Israeli Palestinian context with the Arab citizens in Israel as the GG (similar to the pilot study), and the religious factions in the Israeli context with the Liberal Religious Jewish community as the GG^2^.

In line with the GG hypothesis, and in both contexts, the more participants viewed the intermediate group as having a dual identity, the more positive their attitudes toward the outgroup became^3^. Given these findings, we proceeded to test the GG hypothesis experimentally (to increase internal validity) and assess improvement of intergroup relations through an actual resource allocation task, which is a behavioral measure.

Specifically, we conducted two experiments. The first experimental study aimed to test whether the presence of a GG that clearly encompasses multiple identities, would improve intergroup behavior under highly controlled settings. On-line participants were first assigned to artificially created groups, based on personal inconsequential preferences (Tajfel, 1978), and the key outcome was the amount of resources they allocated to the outgroup vs. the ingroup. In the control condition, the groups were created in a dichotomous manner, reflecting a more traditional two-group context. In the experimental condition, the groups were created such that there was an ingroup, an outgroup, and a GG that shared attributes with both the ingroup and the outgroup, and was thus perceived as having a dual identity. According to the GG hypothesis, and to the findings from the Pilot and correlational studies, the perception of multiple identities (i.e., in the experimental condition) should improve intergroup attitudes and behavior, as compared to a control condition. The results of this study supported this prediction: The presence of a GG that encompasses multiple identities led to more positive intergroup attitudes and behavior. Participants in the GG condition, compared to those in the control condition, allocated more resources to the outgroup, had greater contact motivation, and showed higher tendency for equal division and a lower tendency for complete discrimination.

The second experimental study aimed to replicate the first one while adding two additional elements: First, the experiment was carried out in small groups as opposed to individually^4^. Second, this study was performed in the lab rather than on-line so that we would have better control over the participants’ environment and thus a better ability to simulate the dual identity condition. The second experimental study replicated the results of the first in a more meaningful and interactive context by showing that the presence of a GG leads to more positive intergroup attitudes and behavior. As in the previous study, and in line with the GG hypothesis, participants in the experimental GG condition collectively allocated more resources to the outgroup, had a higher tendency for equal division, a lower tendency for complete discrimination, and showed greater contact motivation.

However, these experiments used relatively artificial groups, which makes generalization to the real world somewhat difficult. Therefore, we followed-up these studies with another set of two quasi-experiments that tapped into the issue of racism in the United States, and the presence of the biracial GG (Levy et al., under review). The prediction here was that if the presence of a GG were to have the same positive influence in the real world scenario as it did in the artificial group setting, it should reduce prejudice in the form of symbolic racism. To test this hypothesis, we presented the participants with sets of photographs of individuals from different races in the pretext of a memory exercise. In the control condition participants were presented with photographs of Black and White individuals, and in the experimental condition participants were presented with the same photographs but with the addition of biracial individuals’ photographs as well. As expected, the results showed that the presence of a biracial GG significantly diminished racist perceptions, and also improved the attitudes toward outgroup victims of current intergroup conflict events^5^.

In a final study, we directly manipulated the level of perceived GG multiple identity in the Israeli-Palestinian context, by providing the Israeli participants with survey data showing that the majority of the Arab Israeli GG members saw themselves both as Israelis and Palestinians and did not find a contradiction in their dual identification. Then, we examined the effects of that manipulation on behavior and attitudes toward the Palestinian outgroup compared to participants in an empty control condition. Furthermore, this study explored mediating variables in order to try and shed some light on the underlying mechanism of the GG effects.

In line with previous studies, the results showed that participants in the experimental dual identity condition allocated more resources to the outgroup, and they also displayed decreased support for aggressive policies toward the outgroup. Importantly, this study also suggested initial evidence for underlying psychological mechanisms, as the presence of a dual identity led to reduced negative stereotyping of the dual identity group, reduced ingroup identification, and reduced anger toward the outgroup which all in turn predicted improved outgroup orientations. These findings are in line with the GG hypothesis, and suggest that perceiving an intermediary group as dually identified with both the ingroup and the outgroup should have positive effects on intergroup attitudes and behavior (See **Table 1**).

**Table 1:** Empirical studies examining the positive effect of the presence of a perceived multiple identity GG on intergroup relations between its external counterparts (Levy et al., 2017, under review).

Study type	Context	Manipulation	Dependent variables
Pilot	Israeli – Palestinian	N.A.	•Perceptions •Attitudes •Emotions
Correlational	Israeli – Palestinian and Secular – religious	N.A.	•Resource allocation to outgroup •Contact motivation with outgroup
Experimental	Minimal group paradigm	GG presence	•Resource allocation to outgroup •Contact motivation with outgroup
Experimental	Minimal group paradigm	GG presence	•Resource allocation to outgroup •Contact motivation with outgroup
Experimental	Black – White in the United States	GG presence	•Symbolic Racism toward Blacks •Outgroup empathy
Experimental	Israeli – Palestinian	GG multiple identity enhancement	•Resource allocation to outgroup •Support of aggressive policies toward outgroup •Mediators: anger toward outgroup, ingroup identification, GG stereotyping

In sum, the studies described above are the first studies to provide empirical evidence for the positive potential multiple identity GGs can have on the external groups that perceive them as such. These studies show that across several different contexts the presence of a group with multiple identities led the groups that represent the sources of these multiple identities to harbor more positive attitudes toward one another and to display more positive intergroup behavioral patterns. These findings fit well with the positive effects of having multiple identities, as reviewed in the previous section, and suggest that there is much promise in the notion that GGs have a role to play when it comes to improving intergroup relations. However, here too it is important to consider the potentially negative side effects of GGs being perceived as having multiple identities in the context of intergroup relations.

## Negative Outcome of Groups *Being Perceived* as Having Multiple Identities

Based on the theories and empirical evidence mentioned above, the positive potential of externally perceiving GGs as having multiple identities in intergroup relations is evident. On the other hand, it is likely that stressing the connection between a GG and the outgroup can easily become detrimental, especially in the context of severe intergroup conflict. Other than the simple animosity that can arise from association with a negatively perceived outgroup, the link between the multiple identity GG and the proximal ingroup, which has so far been stressed as a positive attribute, might sometimes backfire and result in the multiple identity GG posing barriers to conflict resolution. Nonetheless, despite the intuitiveness of this possible backlash, to our knowledge there is no empirical work to date that has examined this negative aspect of groups being perceived as having multiple identities.

We therefore extend our line of thought here to suggest two possible ways in which GGs can be perceived as barriers rather than bridges. First, this is likely the case where those who perceive GG members to have a multiple identity expect them to side with the ingroup. Although this may seem possible at first sight, it implies that any perceived violation of that expectation of ingroup support may lead external groups to treat GG members with strong suspicion, and as potential traitors. Furthermore, while an outgroup member acting on behalf of the outgroup is predictable, for an ingroup member to act on behalf of the outgroup in the context of intergroup conflict is considered treason, and is perhaps one of the most socially deplorable acts possible. This notion corresponds with the literature on the ‘black sheep effect’ (Marques et al., 1988; Pinto et al., 2010) in which attempts by ingroup members that are perceived as a deviation from group norms are judged and punished even more harshly than similar actions originating from outgroup members. Second, in cases where those who perceive GG members to have multiple identities and expect them to side with the outgroup, such perceivers are likely to associate all the negative attributes linked to the outgroup with the GG as well. This perception is bound to frame the GG as a threat and simply amplify the existing conflict tension and animosity.

In sum, while there is no existing empirical evidence regarding the possible back-lash of GGs being perceived as holding multiple identities, it is likely to assume that such an outcome is possible under certain conditions, and depending on different possible affiliations of the GG.

## Possible Explanatory Mechanisms

So far, we have discussed the possible impact GGs might have on intergroup relations. However, we have not addressed the issue of *why* they have such impact. Several studies offer some insight into possible mechanisms. For example, Gocłowska and Crisp (2014) have examined three necessary conditions needed for dual identity to be able to help foster creativity among those who hold such an identity: (1) The dual identifiers need to develop a deep relationship with the two (or more) groups that they belong to, (2) the dual identifiers need to undergo the process of adaptation to living and functioning in a new group, but at the same time remain identified with their original culture, and (3) the dual identifiers need to experience some distance and dissonance between their host and home cultures. While this research focused on creativity which is not necessarily directly linked to intergroup relations, such variables may play a role in the intergroup context as well.

For instance, in terms of relationships with and adaptation to the different groups (condition 1&2 above), Hornsey and Hogg (2000) found that the salience of the different groups representing the multiple identities can affect intergroup bias, and that members of groups with shared multiple identities for whom both categories are salient exhibit lower levels of intergroup bias compared to those for whom only one of the groups is salient. In terms of distance and dissonance between identities (condition 3 above), research by Simon et al. (2013) has examined the aspect of dissonance between identities, or more specifically incompatibility of identities, and has found that incompatible identities among dual identifying immigrants in Germany led to elevated sympathy for political radical action. On the other hand, research by Chayinska et al. (2017) found that compatibility between multiple identities encourages and legitimizes collective action. Another element related to the dissonance between identities that may have intergroup implications, is the level of projection of a single identity on the superordinate inclusive identity which the GG are associated with. Based on the work done on the projection model (Mummendey and Wenzel, 1999; Kessler et al., 2010) the more the multiple identity GG resembles the identity projected on the superordinate identity, the better its chances of evading prejudice and negative emotions.

Additional aspects that have been found to influence the impact of multiple identification in the intergroup context are group status, size, and threat. Some of the positive effects associated with multiple identity in the intergroup context, such as intergroup bias reduction, have been found to only take place among minority groups (González and Brown, 2006), and in contrast, research in a different context has shown that dual identification was most efficient in reducing intergroup bias among high status dual identifying groups (Dovidio et al., 2009). Finally, research by Baysu et al. (2011) has found that in the presence of identity threat, dual identity can be detrimental, but in the absence of such threat dual identity is preferable to any other form of minority identification such as assimilation or separation.

In terms of the impact that multiple identity might have on external groups that perceive a GG as having multiple identities, the recent empirical studies mentioned above have begun to shed some light on the possible mechanism at hand. These studies have found that the presence of a dual identity led to reduced negative stereotyping of the dual identity group, as well as reduced ingroup identification (Levy et al., 2017). Based on these findings, it seems that the impact of the presence of multiple identity GGs on groups that perceive them as such, may be mediated by fostering a more complex perception of social categorization in general.

In sum, it seems that several different mechanisms can explain the potential impact of GGs on intergroup relations. These mechanisms include variables such as: Compatibility or similarity between the multiple identities held by the GG; the type of relationship the GG has with its counterparts; the status and size of the GG compared to its counterparts; the levels of threat felt by and from the GG; the social identification of both the GG and its counterparts; and the manner in which the GG is perceived by the groups it interacts with. While the existing research refers to most of these variables, the literature still lacks a clear model that factors in these elements, and enables a clear prediction of a GG’s impact in different scenarios. Below we will offer a few options of future research that should assist in the formulation of such a model.

## Questions for Future Research

Based on the framework described above, it is evident that research has found both negative and positive impacts of multiple identity GGs in the context of intergroup relations. However, it is mostly unclear *when* one might expect GGs to function as bridges or barriers that improve or worsen intergroup relations. The explanatory mechanisms mentioned in the previous section provide initial explanations as to how GGs might affect intergroup relations, but do not provide a substantial way of predicting when the presence of a GG will be beneficial, and when it might backfire and deteriorate intergroup relations. For us, this question is one of the most important ones that future research should address.

So far, the literature offers mixed results, such as in the case of the projection model (Mummendey and Wenzel, 1999) and subgroups model (Hornsey and Hogg, 2000). Following these two lines of thought, when one of the identities at hand significantly differs from the superordinate identity, and both identities are made salient, the work done by Hornsey and Hogg would probably predict lower levels of prejudice, while the projection model (Mummendey and Wenzel, 1999) would predict the contrary. Similar contradictory findings appear in the research on the effects of dual identity’s interaction with group size and status mentioned above. While González and Brown (2006), found their effect mainly among dual identifying minority groups, Dovidio et al. (2009) found similar effects mainly among high status dual identifying groups. This suggests that there might be a more significant predictor of the type of impact GG have on intergroup relations, or alternatively, that a combination of several variables add up to affect the impact of GGs either positively or negatively.

Accordingly, future research should explore the elements that have been found as influential in affecting the type of impact GGs have on intergroup relations. For instance, status and size of the GG has been found to interact with the GG’s influence on intergroup bias reduction, but the manner in which these variables interact is still unclear (González and Brown, 2006; Dovidio et al., 2009). Therefore, future research should focus on GG status and size as possible moderators and examine what conditions are necessary in this regard for intergroup bias reduction. Additionally, based on the conceptual work described above it is likely that sense of threat from the GG might inhibit the GG’s ability to facilitate conflict resolution. Future research dealing with multiple identities and GGs should test for threat as a possible mediator, and check if being threatened by the GG diminishes its positive impact on intergroup relations. Research examining variation in such variables should enable the construction of a detailed model describing the conditions for the different possible outcomes, and would greatly improve the ability to predict the optimal way to manage such identities in the context of intergroup dynamics. Moreover, such a model would also enable the development of practical implementations of the GG potential^6^.

Besides the specific variables described above there are also broader questions related to the GG prospect that call for additional research. As we have proposed, at this point in time it seems best to approach the issue of GGs while maintaining the distinction between how such groups identify themselves, and how these groups are perceived by others (Kang and Bodenhausen, 2015). While the work done in the realm of how these multiple identities affect the GG themselves is more substantial, research on how GG that hold multiple identities affect the other groups involved has only just began scratching the surface. Moreover, as a result of the distinction between how individuals in GGs experience their identity, and how these groups are perceived by others, there is the possibility that these two perspectives might not always be aligned. It is likely that when both the GG itself and its counterparts all perceive the GG as holding multiple identities, then the presence of the GG will impact intergroup relations as described above. However, it is not clear what would happen in cases in which the GG is perceived as holding multiple identities, but the GG itself only identifies with one group. Accordingly, future research should examine the outcomes of such dissonance, both on the GGs and on their counterparts as well.

Due to the lack of empirical research on the possible negative outcomes in the context of GGs being perceived as having multiple identities by others, future research should start studying this gap in the literature. As described above, the expectation of GG members to side solely with the ingroup, might perceive the GG’s interaction with the outgroup as an act of betrayal. Moreover, affiliation of the GG with the outgroup coupled with mistrust are likely to have a negative intergroup impact as well. Accordingly, future research should examine expectation of GG loyalty, as well as elements of trust and threat, in order to account for possible GG backlash effects.

An additional aspect that may play a significant part in the integration of multiple identities into the intergroup relations framework is the GG motivation to take on the responsibility of facilitating the intergroup relations among its different counterparts. It is likely to assume that GGs might have varying levels of motivation to take on, or shy away from, the role of intergroup facilitation. The underpinnings of these motivations should be explored and mapped out in order to enhance the ability to predict GG action.

Another noteworthy research topic that has yet to be addressed is the development of novel identities among groups holding multiple identities. So far, the literature in the field has addressed groups of people with shared multiple identities as maintaining different existing identities coupled together. However, it is possible that for individuals and groups that maintain a state of multiple identities over time, the multiple identities morph into a new single identity that is distinct and independent from its identities of origin (Rockquemore, 1998; Simon and Ruhs, 2008). This notion may account for some of the variation in the existing literature regarding multiple identities. Indeed, in some instances individuals who hold multiple identities, may in reality be holding a new fused single identity.. Moreover, the presence of multiple identities (e.g., as reflected in the GG empirical studies) might play a role in affecting intergroup relations simply as a result of exposure to complex identity structures. This would mean that exposure to any group with multiple identities would have the same effect on intergroup relations regardless of its connections to the conflicting groups at hand. Both these options contend with the notion presented in this paper by either eliminating the uniqueness of GGs, or by extending that uniqueness to any multiple identity group regardless of its affiliation, and require further research.

Finally, while almost all the research described in this paper is from the realm of the social psychology of intergroup relations, there are several disciplines that have a vested interest in multiple identity GGs as well. For example, researchers in the field of social cognition have developed an approach in which social categories are perceived in a continuous manner (Maddox, 2004; Eberhardt et al., 2006). Based on the racial GG studies presented above, it seems likely that the perception of racial categories as continuous may have the potential to alleviate racist behavior, and that the presence of racial GGs have the potential of emphasizing this continuum. Future research should examine both these assumptions that may lead to new and innovative ways to combat racism. Another possible interdisciplinary connection is with the work on social networks, from the fields of sociology and communications. This work has great potential to enrich our understanding of the role GGs have to play in intergroup relations. Research in these fields has found that strategically situated groups such as the GG can be expected to facilitate the dissemination of information between conflicting social groups, to create interpersonal connections across social fault lines, and to induce efficient communication between groups in conflict (Granovetter, 1973; Long et al., 2013; Centola, 2015; Repke and Benet-Martínez, 2017). Accordingly, an interdisciplinary approach might prove very useful in this regard.

## Conclusion

When it comes to social-psychological theories regarding groups of people with shared multiple identities in intergroup relations, it seems that different theories only address specific facets of the potential role these groups can play in intergroup dynamics. Our GG framework builds on several of the existing theories and extends them by outlining *what* functions multiple identity GGs may serve (as *bridges* or *barriers*), and *how* their presence and/or experience may improve or deteriorate intergroup relations. As such, it also provides a research agenda along those lines, as well as additional important questions such as *when* GGs can be expected to serve as bridges or barriers.

Importantly, our review of relevant published and unpublished findings suggests that multiple identity GGs hold a unique potential when it comes to improving intergroup relations. GGs which can potentially be situated interchangeably in regard to a given social border, can act as catalysts for the attempts to shift or redefine the borders between social categories, and the mere presence of a GG in situations of intergroup conflict can be expected help partially dismantle social categories that otherwise facilitate intergroup strife. GGs multiple affiliation might signal to their respective groups that a superordinate identity, incorporating both groups, is possible. Additionally, the manner in which GGs cross social categories can help confront stereotypical and heuristic modes of thinking, and raise tolerance for outgroups in general. These positive effects multiple identity GGs may have on intergroup relations originate both from such groups perceiving themselves as having multiple identities, and from such groups being perceived as having multiple identities by their social counterparts. However, the fleshing out of these multiple identities and social affiliations of GGs can also be detrimental under certain circumstances. Hopefully, future research will shed more light on the double-edged sword of multiple identities, and tease apart the factors that induce negative intergroup outcomes from those that promote positive intergroup dynamics and facilitate intergroup conflict resolution.

## Author Contributions

All authors substantially contributed to the conception of the work; and drafted the work; and approved the version to be published; and agreed to be accountable for all aspects of the work.

## Conflict of Interest Statement

The authors declare that the research was conducted in the absence of any commercial or financial relationships that could be construed as a potential conflict of interest.

## Footnotes

1 This structure also corresponds with other reviews dealing with different aspect of groups with multiple identities, such as the work done by Kang and Bodenhausen on social cognition.

2 The Liberal Religious Jewish community in Israel represents a group that is situated in between two separate, often conflicting, categories: Secular Jews, who are a relatively standard westernized secular society, and Ultra-Orthodox Jews who are a zealous religious society which adamantly separates itself from the non-religious Jewish world in all walks of life. On the one hand, Liberal Religious Jews still abide to Jewish religious law but are, on the other hand, immersed in their local secular cultures as well. This enables them to be identified with both Secular and Orthodox societies, and possibly act as a gateway between them. The participants in the study were Secular (ingroup), and the outgroup was the Ultra-Orthodox community.

3 The attitudes toward the outgroup in this study were measured by contact motivation with the outgroup, general feelings toward the outgroup, and also a hypothetical resource allocation between the ingroup and the outgroup.

4 Studies have shown that intergroup interactions are generally more competitive than interpersonal interactions (Insko et al., 1992; Wildschut et al., 2003). This suggested that allowing individuals to make allocation decisions in groups would put them in a stronger intergroup conflict situation thus enabling us to increase conflict intensity without losing experimental control.

5 As opposed to the previous studies in which the described effects were found across the board, in this study, participants that were not prone to racism (low SDO) did not differ as a function of whether biracials were present. However, among participants prone to racism (high SDO) we found a replication of the previous GG studies in which the presence of a biracial GG significantly diminished racist perceptions, and also improved the attitudes toward outgroup victims of current intergroup conflict events.

6 For example, *Extended contact* might be further developed as a conflict resolution tool based on the findings of this research. The extended contact hypothesis contends that knowledge about cross-group friendships (i.e., knowing that an ingroup member has a positive relationship with an outgroup member) can improve outgroup attitudes and thus advocates the advantages of intergroup contact by proxy (Wright et al., 1997; Christ et al., 2014). Usually, in order for extended contact to have a positive effect, the process has to entail an ingroup exemplar having contact with an outgroup exemplar (Wright et al., 1997). Nonetheless, although dual identifiers are not classic ingroup exemplars, the frequency of their unmediated interaction with both counterparts holds the potential for effects similar to the ones observed in the extended contact literature.
